# COVID-19 Vaccine Perceptions Survey for Real-Time Vaccine Outreach in Marin County, California

**DOI:** 10.7759/cureus.36583

**Published:** 2023-03-23

**Authors:** Jasmine Soriano, Haylea Hannah, Karina Arambula, Tyler Evans, Rochelle Ereman, Matthew Willis

**Affiliations:** 1 Epidemiology and Public Health, Marin County Department of Health & Human Services, San Rafael, USA; 2 Population and Public Health Sciences, Keck School of Medicine of USC (University of Southern California), Los Angeles, USA

**Keywords:** vaccination, survey, public health, equity, vaccine hesitancy, covid-19 vaccine, covid-19

## Abstract

Background: Understanding and addressing coronavirus disease 2019 (COVID-19) vaccine hesitancy is crucial to informing vaccination outreach strategies and achieving high vaccination coverage. Marin County, California, United States, has a history of vaccine hesitancy regarding childhood vaccinations required for school entry.

Objectives: We aimed to describe and address COVID-19 vaccine hesitancy in Marin County to inform outreach and messaging. Our objectives were to identify subgroups with high COVID-19 vaccine hesitancy early in distribution, better understand local concerns and feedback about the COVID-19 vaccine distribution process, and inform tailored vaccine messaging to increase vaccination confidence and coverage.

Methods: The survey, which was administered from January 3 to May 10, 2021, queried demographics, vaccine acceptance, reasons for hesitancy, and reasons for acceptance. Open-ended questions were used for respondents to report additional reasons for hesitancy and for general feedback about the vaccine distribution process. We conducted quantitative and qualitative analyses stratified by COVID-19 vaccine acceptance to identify subgroups with high hesitancy. Results were shared weekly in real-time with leadership and key community partners working on vaccine outreach.

Results: Among the 5,618 survey responses, there were differences in vaccine hesitancy by sociodemographic characteristics with the highest hesitancy reported among subgroups identifying as Black/African American and young adult, and within the lowest family income grouping. The most common reason for vaccine hesitancy was “uncertain about the side effects of the vaccine” (67.3% endorsement) and responses varied by race and ethnicity. Qualitative data revealed equity-related, vaccine distribution, and vaccine access themes that were not present in structured responses. Vaccine hesitancy survey results were paired with vaccination coverage and COVID-19 case data to inform tailored outreach strategies and priorities week-to-week.

Conclusions: Marin County had some of the highest COVID-19 vaccination rates in the United States during the pandemic and met equity goals aimed at ensuring vulnerable populations received vaccinations. Presenting real-time survey findings with leadership and key community partners informed a timely and tailored COVID-19 vaccine outreach and delivery strategy.

## Introduction

Marin County, population 259,943, is located in California’s San Francisco Bay Area [1]. While Marin is characterized by long life expectancy and high average income, opportunities for health are not evenly distributed; Marin also ranks first for the greatest racial disparities in the state [2]. For this reason, Marin County Health & Human Services (HHS) prioritizes racial equity in prevention, outreach, and service delivery operations [3].

Marin has a history of vaccine hesitancy with the highest rates of Personal Belief Exemptions from school-required vaccinations among Bay Area counties in 2015 [4]. In the past, community-wide surveys served as a useful resource for Marin County HHS to evaluate changing attitudes towards mandatory vaccinations required for school entry. Recognizing that the reasons for coronavirus disease 2019 (COVID-19) vaccine hesitancy may differ from those for other vaccines, we used this framework to understand perceptions towards the COVID-19 vaccine with the ultimate goal of informing vaccination outreach. Because of the severe outcomes associated with COVID-19 infection paired with limited resources to produce a new vaccine rapidly, vaccine roll-out had to be equitable, efficient, informed, and efficacious in protecting those most vulnerable to severe outcomes associated with COVID-19.

Access to the COVID-19 vaccine was impeded by the pandemic conditions of the time and pervasive mistrust of public health and medical institutions [5]. Research among different populations has shown that COVID-19 vaccine attitudes can vary by race/ethnicity [6], age [6-8], neighborhood [9], political affiliation [6,10], socioeconomic status [6,8,11], and government trust level [6,11-13]. Knowing that vaccination acceptance is core to vaccine coverage [14], and efficient and equitable vaccine coverage is core to preventing COVID-19 [5], we launched a community-wide COVID-19 vaccine perceptions survey. The purpose of our survey was to identify local subpopulations with high vaccine hesitancy for real-time, targeted outreach, and to better understand reasons for hesitancy for tailored messaging that would improve vaccine acceptance and trust. COVID-19’s disproportionate burden among Black/African American and Hispanic/Latino/a/x populations [15-17] amplified the importance of focusing on equity throughout vaccine outreach and delivery in Marin County.

## Materials and methods

Survey design

We developed a survey to be conducted among people who lived or worked in Marin County. Survey questions included race, ethnicity, age, educational attainment, household income, occupation, geography, and acceptance of the COVID-19 vaccine. Validated items were included when possible. Respondents were classified as hesitant (“No” or “Unsure”) or accepting (“Yes”) based on their response to “Are you open to receiving the COVID-19 vaccine?” For those who were hesitant, we posed structured questions regarding reasons for hesitancy and what might lead them to receive the vaccine. For those who were accepting, we asked reasons for acceptance. Open-ended questions probed additional reasons for hesitancy, what would make them comfortable receiving the vaccine, reasons for acceptance, and other comments. The University of Washington Internal Review Board’s policy asks that researchers self-determine that a project does not meet criteria for human subjects research and is thus exempt from IRB review. Their self-determination tool indicated that this project did not meet criteria for human subjects research.

Survey administration

Survey responses were collected electronically via Google Forms (Google LLC, Mountain View, California, United States) from January 3 to May 10, 2021. Responses were solicited through email, text, community partners, town hall presentations, the County COVID-19 website, and an in-person event conducted orally in Spanish. The survey was available in English and Spanish.

Quantitative analysis

We compared the survey sample’s demographic distribution with Marin’s general adult population (18+ years). We used proportions and chi-square tests to analyze responses by race/ethnicity, age, household income, educational attainment, city of residence, and occupation. Subgroups with fewer than 30 respondents were combined or excluded from analyses. We compared endorsement of reasons for COVID-19 vaccine hesitancy by racial and ethnic subgroups. All data were analyzed using Stata/SE 16.0 (2019; StataCorp LLC, College Station, Texas, United States) [18].

Qualitative analysis

Qualitative methodology was used to identify COVID-19 vaccine perceptions that had not been anticipated and were not included in structured response options. We used a modified grounded theory approach to identify main themes present in the open-ended, free-text survey responses [19]. Open-ended survey questions asked respondents to provide reasons they were hesitant or accepting of the COVID-19 vaccine and any additional feedback they would like to share. Because these questions were not structured to receive a particular response, we utilized a grounded theory methodology to allow for themes to naturally emerge from the data. First, two independent coders identified preliminary themes from open-ended survey responses in English and Spanish. Coders reconciled these preliminary themes to develop a common set of final themes. Lastly, coders identified keywords and combinations of keywords within the open-ended responses that were associated with each theme. These keywords were used to assign all open-ended responses to their associated themes. Responses could have more than one theme assigned to them. Final themes were tabulated to observe their prevalence. We constructed a conceptual model to convey themes of vaccine hesitancy and acceptance and the relationships between them. All themes presented reflect those identified from both English and Spanish language surveys.

Sharing findings to inform vaccination strategy

**Figure 1:**
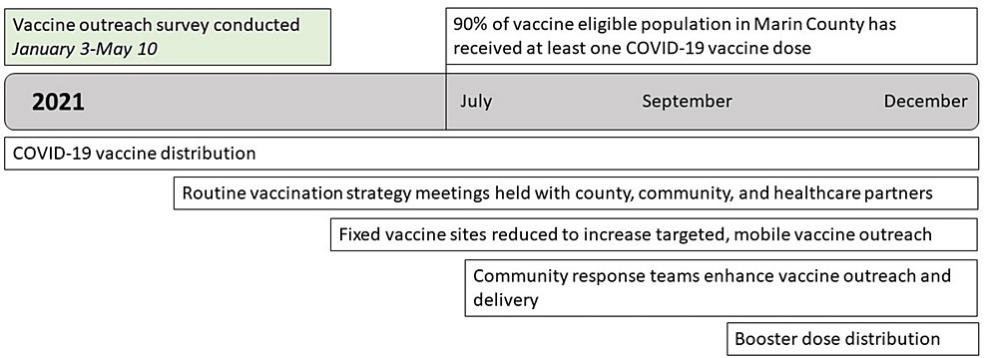
Timeline of COVID-19 Vaccination Perceptions Survey and Outreach Activities in Marin County, California, 2021 COVID-19: coronavirus disease 2019

COVID-19 vaccine distribution began in Marin County in January 2021 and continued throughout the year (Figure 1). Also beginning in January, Marin County HHS hosted weekly Vaccination Strategy Meetings with Public Health leadership, community partners, and local healthcare entities. Intermediate survey findings were shared during these weekly meetings. During data collection, findings were also shared via ad hoc oral presentations and in real-time using a dashboard created in Infogram, a data visualization tool. Findings shared included subgroups with the highest vaccine hesitancy, reasons for vaccination hesitancy and acceptance by race and ethnicity subgroups, and themes about equity, logistics, and vaccination decision-making from qualitative data.

As Marin County reached their target goal of 90% of their eligible population receiving at least one dose of the COVID-19 vaccine, resources distributed to fixed vaccination sites were reduced to increase those available for targeted, mobile vaccine distribution. This approach was further enhanced with the development of four geographically-based community response teams that supported vaccine distribution and other pandemic response activities. Survey findings were paired with COVID-19 vaccination coverage data by demographics (age group, race/ethnicity) and census tract to inform real-time vaccine outreach strategies countywide and for the community response teams (Figure 1).

## Results

From January 3 through May 10, 2021, we received 5,618 survey responses; 5,429 (96.6%) in English and 189 (3.4%) in Spanish. Non-Hispanic White individuals were overrepresented among respondents (76.6%) when compared with Marin’s adult population (69.0%). There were significant differences (p < 0.05) in COVID-19 vaccine hesitancy by race/ethnicity, age, educational attainment, household income, and city of residence (Table 1). Subgroups with the highest hesitancy identified as Black/African American (27.5%), between 19 to 34 years of age (14.7%), earning a household income of less than $25,000 (8.6%), obtaining some college education level (8.1%), and residing in Marin City (28.7%), a Marin community that is a historically significant Black/African American cultural center (Table 1).

**Table 1:** Factors Associated with COVID-19 Acceptance or Hesitancy in Marin County, California *Subgroups with the highest hesitancy within each characteristic BA: Bachelor of Arts; BS: Bachelor of Science; COVID-19: coronavirus disease 2019

Characteristic	Accepting n (%)	Hesitant n (%)	p-value
Total Respondents	5314 (95.1)	271 (4.9)	
Race/Ethnicity			<0.05
Hispanic/Latino/a/x	389 (91.3)	37 (8.7)	
White	2607 (97.3)	73 (2.7)	
Black/African American*	58 (72.5)	22 (27.5)	
Asian	147 (94.2)	9 (5.8)	
Other	142 (89.9)	16 (10.1)	
Age			<0.05
0-18	40 (100.0)	0 (0.0)	
19-34*	396 (85.3)	68 (14.7)	
35-49	952 (92.9)	73 (7.1)	
50-64	1601 (95.5)	76 (4.5)	
65-79	1964 (98.2)	35 (1.8)	
80+	313 (98.7)	4 (1.3)	
Household Income			<0.05
Less than $25,000*	310 (91.4)	29 (8.6)	
25-49	447 (94.1)	28 (5.9)	
50-99	893 (96.5)	32 (3.5)	
100-149	833 (96.5)	30 (3.5)	
150-199	591 (97.8)	13 (2.2)	
200 or More	1318 (97.0)	41 (3.0)	
Educational Attainment			<0.05
High school or less	342 (93.4)	24 (6.6)	
Some College*	703 (91.9)	62 (8.1)	
Associate's Degree	316 (93.7)	25 (7.3)	
BA/BS Degree	1676 (96.3)	64 (3.7)	
Graduate/Professional Degree	2090 (97.1)	63 (2.9)	
City of Residence			<0.05
Belvedere Tiburon	77 (93.9)	5 (6.1)	
Corte Madera	165 (99.4)	1 (0.6)	
Fairfax	155 (94.5)	9 (5.5)	
Greenbrae	146 (98.6)	2 (1.4)	
Kentfield	99 (98.0)	2 (2.0)	
Larkspur	149 (97.4)	4 (2.6)	
Marin City*	67 (71.3)	27 (28.7)	
Mill Valley	812 (95.9)	35 (4.1)	
Novato	907 (96.3)	35 (3.7)	
Ross	39 (100.0)	0 (0.0)	
San Anselmo	373 (98.7)	5 (1.3)	
San Rafael	1075 (97.0)	33 (3.0)	
Sausalito	157 (94.6)	9 (5.4)	
Tiburon	140 (97.9)	3 (2.1)	

Among respondents who identified as vaccine hesitant, the two most endorsed reasons for hesitancy were “Uncertain about the side effects of the vaccine” (67.3%) and “I feel the vaccine approval process was too quick” (57.7%). These endorsements varied by racial and ethnic subgroup. Eighty-one percent of White vaccine-hesitant respondents were uncertain about the side effects of the vaccine while 66% of their Hispanic/Latino/a/x counterparts endorsed this reason. Sixty-eight percent of respondents who identified as Black/African American felt the vaccine approval process was too quick compared to 61% among White and 50% among Asian vaccine-hesitant respondents.

When asked when hesitant individuals would be more comfortable receiving the vaccine, the most endorsed responses were “I know more about the vaccine’s side effects” (50.5%), and “More time passing” (50.2%). Among respondents who did not identify as vaccine hesitant, the two reasons for vaccine acceptance with the highest endorsement were “I want to protect myself from getting COVID-19” (87.1%), and “I want to protect my family and community from getting COVID-19” (86.0%). Among the 62 open-ended responses to other reasons for vaccine hesitancy, leading themes identified were distrust in institutions (n=12), concerns over long-term reactions to vaccines (n=10), the vaccine being too new or approved too quickly (n=10), and general feelings of fear (n=10). An additional important theme identified was mistrust of governmental institutions for fear of harming people of color (n=2), which was supported by Black/African American and American Indian/Alaskan Native respondents.

**Figure 2:**
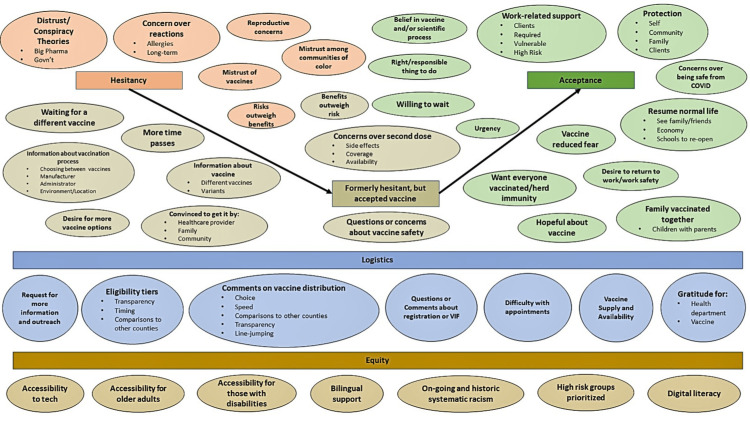
Conceptual Model of COVID-19 Vaccine Hesitancy, Acceptance, and Contributing Factors in Marin County, California COVID-19: coronavirus disease 2019

The conceptual model developed from open-ended responses shows the spectrum of vaccine hesitancy to acceptance, highlighting factors that might influence hesitant individuals to accept the vaccine and affect vaccine access (Figure 2). Some participants also reported eventually being convinced to get the vaccine by their healthcare provider, family, and community.

Several open-ended responses supported themes around challenging logistics that made it difficult for people to get the COVID-19 vaccine. These themes included requests for more information and outreach, a desire for more transparency on vaccine distribution, difficulty finding vaccine appointments, and comments about who was eligible to receive the vaccine during early vaccine delivery. Lastly, several respondents supported equity-related themes such as accessibility to technology, for older adults, and those living with disabilities, and more support for non-English speakers.

Findings were shared in the weekly Vaccination Strategy Meetings that included County leadership, healthcare partners, and other community-based organizations participating in COVID-19 vaccine outreach (Figure 1). In addition to survey findings, the County’s Epidemiology team shared data on vaccination rates by demographic and geographic subgroups. These near real-time data informed the coordinated approach of vaccine outreach and delivery while considering hesitancy as one contributor to vaccine coverage. Findings were shared with healthcare and community-based organization partners to provide targeted, tailored outreach to the community. Geographic data informed decisions for the County’s mobile vaccination unit locations. Qualitative findings in particular were used to address concerns about logistics, develop COVID-19 vaccine resources, and tailor communication to subpopulations with high hesitancy.

## Discussion

Among our survey respondents, subgroups with the highest COVID-19 vaccine hesitancy included underrepresented and lower socioeconomic status groups and regions in Marin County. Higher hesitancy was observed in the Black/African American subpopulation than in other race/ethnicity groups. Additionally, younger adult age groups (19 to 34 years of age) were more vaccine hesitant than older age groups. Similar patterns were seen in other populations [6-8,11,15]. Collecting reasons for vaccine hesitancy, what could build comfort among hesitant individuals, and reasons for acceptance in both structured and unstructured questions allowed us to share specific perceptions by subpopulation. With frequent presentations and a live dashboard shared throughout this crucial time in early vaccine delivery, stakeholders were able to use the survey data combined with vaccination coverage data to make equity-focused vaccine strategy decisions.

Much like in other studies, concerns about side effects, the novelty of the vaccine, and distrust of institutions were apparent in our quantitative and qualitative findings [11-13,18-19]. However, these concerns varied among people from different racial and ethnic subgroups, which may imply that COVID-19 vaccine messaging and information could be tailored to prevalent concerns in the community. For example, Black/African American vaccine hesitant respondents were more likely to agree that the COVID-19 vaccine approval process was too quick. This informed the creation of messaging about the development of the vaccine. These data stressed the importance of increased collaboration between Marin County HHS and community-based partners for coordinated communication with the public. Marin’s Public Health Officer (and deputies) frequently shared local COVID-19 vaccination data publicly including statistics about adverse events via public forums, status updates, and tailored communications [19]. Marin County overcame their reputation as a vaccine hesitant community when they were among the counties with the highest vaccination coverage in the United States during the COVID-19 pandemic [20]. Vaccine outreach materials developed by the Public Information Officer addressed apprehensions and questions that were revealed through survey data. The efforts to understand vaccination hesitancy and acknowledge community concerns during a mass vaccination campaign may have resulted in improved trust from a formerly hesitant community.

This study emphasizes the importance of Marin County HHS continuing to build trust with the community by integrating local data into public health strategy. Other research supports the importance of identifying and addressing community concerns about the COVID-19 vaccine early in vaccine distribution [12,21]. Survey data allowed us to better understand opinions of the COVID-19 vaccine and address misinformation at the crucial time of vaccine roll-out. Other jurisdictions can use recommendations to collect local data and collaborate with healthcare and community-based partners to drive public health efforts. Additional research could include COVID-19 vaccine perception studies in other populations as well as follow-up studies in Marin about other health advancements and promotion efforts. Marin County HHS has already conducted a community-based assessment of the effectiveness of public health communications.

Strengths and limitations

An asset of this project was the utility of the data for timely public health and healthcare action. The communication of survey findings through several formats early in COVID-19 vaccine delivery allowed decision-makers to easily digest and incorporate community perspectives into vaccine strategy. Perhaps in part due to its proactive and innovative uses of data for public health action like this survey, Marin County had the highest COVID-19 vaccination rates in California during the summer of 2021 [20,22-23].

Another strength of this survey was the use of qualitative data. Because the survey was administered early in COVID-19 vaccine delivery, there was little known about the reasons for vaccine hesitancy and acceptance. Therefore, structured response options designed in December 2020 did not necessarily capture the concerns that would change and arise over time. Through analyzing open-ended responses with a modified grounded theory approach, we learned important, new local perspectives that were incorporated into vaccine outreach materials and dialogue. The conceptual framework highlighted important factors among respondents whose confidence in the vaccine grew and allowed us to address hesitancy as it related to race and ethnicity (Figure 2). Mixed methods study designs that pair qualitative and quantitative data to understand a health issue offer an opportunity for each type of data to complement the limitations of the other and allow for sharing of different perspectives [24].

The timeframe in which we conducted the survey was both beneficial because of its pivotal position in early vaccine delivery during a surge in COVID-19 infections, and impacted the design of the survey and the interpretation of our findings. During survey development, COVID-19 vaccine perspectives were not widely available and attitudes were likely to change over time. Therefore, the reasons for hesitancy and acceptance were anticipated based on limited information. While open-ended responses helped gather opinions not in structured response options, we may have missed or underestimated local concerns in the later stages of the pandemic, such as those related to distrust. Due to political, demographic, and cultural factors, COVID-19 vaccine perceptions may differ greatly by community. While these local data informed practice in Marin County, generalizing these findings to other settings should be done with caution. This emphasizes the value of collecting data locally in order to drive effective public health efforts.

Another limitation of the study was the sampling approach. Emailing the survey link to healthcare employees when they were eligible for vaccination and posting it on the County’s COVID-19 vaccine website were two administration methods that could have resulted in an overrepresentation of survey respondents who were more likely to be highly educated or more digitally literate than the general population. We may have failed to reach key populations, such as those who speak languages other than English or Spanish, live with disabilities, or lack computer access. These groups could have different concerns about the COVID-19 vaccine than survey respondents. To capture responses from additional subpopulations, we hosted an in-person interview event and advertised the survey in a town hall. Even with these efforts, our survey distribution did not yield a sample representative of the Marin population by important characteristics such as race and ethnicity.

## Conclusions

The Marin COVID-19 Vaccine Perceptions Survey was an example of using local, timely, community-generated data to iteratively shape public health approaches during a pandemic. Survey responses allowed us to identify subgroups with higher hesitancy and understand local concerns through structured and unstructured data during a pivotal time in which the novel vaccine was being distributed. The data were used in near real-time to inform vaccination strategy and messaging. These data were paired with vaccination coverage and COVID-19 incidence data to acknowledge hesitancy as well as access, infection risk, and coverage during vaccine distribution. This applied approach may have contributed to an exceptionally high COVID-19 vaccination coverage in Marin County, despite its history of vaccine hesitancy. Marin County will continue to shape public health strategy and build community trust through learnings from local data.
